# COVID-19 Sepsis and Microcirculation Dysfunction

**DOI:** 10.3389/fphys.2020.00747

**Published:** 2020-06-26

**Authors:** Antonio Colantuoni, Romeo Martini, Patrizia Caprari, Marco Ballestri, Pier Leopoldo Capecchi, Agostino Gnasso, Rosalia Lo Presti, Antonella Marcoccia, Marco Rossi, Gregorio Caimi

**Affiliations:** ^1^Department of Clinical Medicine and Surgery, School of Medicine and Surgery, University of Naples Federico II, Naples, Italy; ^2^Unit of Angiology, Department of Cardio Thorax and Vascular Sciences, Azienda Ospedaliera Universitaria Padova, Padua, Italy; ^3^Istituto Superiore di Sanità (ISS), Rome, Italy; ^4^Nephrology Dialysis and Transplant Unit, University Hospital of Modena, Modena, Italy; ^5^Department of Medical, Surgical and Neuroscience Sciences, University of Siena, Siena, Italy; ^6^Department of Clinical and Experimental Medicina, Magna Graecia University of Catanzaro, Catanzaro, Italy; ^7^Department of Psychology, Educational Science and Human Movement, University of Palermo, Palermo, Italy; ^8^UOD Vascular Medicine and Auto-immunity, Sandro Pertini Hospital, Rome, Italy; ^9^Dipartimento di Medicina Interna, Università di Pisa, Pisa, Italy; ^10^Department of Science for Health Promotion and Mother to Child Care, University of Palermo, Palermo, Italy

**Keywords:** COVID-19, microcirculation, endothelial cells, angiotensin II, thromboxane (TxB2)

## Abstract

The spreading of Coronavirus (SARS-CoV-2) pandemic, known as COVID-19, has caused a great number of fatalities all around the World. Up to date (2020 May 6) in Italy we had more than 28,000 deaths, while there were more than 205.000 infected. The majority of patients affected by COVID-19 complained only slight symptoms: fatigue, myalgia or cough, but more than 15% of Chinese patients progressed into severe complications, with acute respiratory distress syndrome (ARDS), needing intensive treatment. We tried to summarize data reported in the last months from several Countries, highlighting that COVID-19 was characterized by cytokine storm (CS) and endothelial dysfunction in severely ill patients, where the progression of the disease was fast and fatal. Endothelial dysfunction was the fundamental mechanism triggering a pro-coagulant state, finally evolving into intravascular disseminated coagulation, causing embolization of several organs and consequent multiorgan failure (MOF). The Italian Society of Clinical Hemorheology and Microcirculation was aimed to highlight the role of microcirculatory dysfunction in the pathogenetic mechanisms of COVID-19 during the spreading of the biggest challenges to the World Health.

## Introduction

The spreading of Coronavirus (SARS-CoV-2) pandemic, known as COVID-19, has caused a great number of fatalities all around the World (World Health Organization, 2020). Up to date in Italy we had more than 28,000 deaths, while there were more than 205.000 infected (Ministero della Salute, 2020 May 6).

In most patients COVID-19 induced symptoms characterized by weakness or fatigue with cough and fever; in some cases, diarrhea, and vomiting with arthralgia were reported. More than 15% of Chinese patients progressed into severe complications, with acute respiratory distress syndrome (ARDS), needing intubation and emergency treatment (Chen N. et al., 2020; Guan et al., 2020). However, a real estimation of the percentage of patients treated in intensive care units (ICU) and the overall percentage evaluation of the death rate by COVID-19 require an accurate assessment of the number of people infected in each Country: these data are lacking at moment. Several reports have tried to clarify the pathophysiology of COVID-19 and the number of papers is exponentially growing. Significant cytokine storm (CS) and coagulation alterations have been described in critical patients, as previously reported in patients with sepsis and ARDS (Zhang W. et al., 2020). However, in a very high percentage of dead patients by COVID-19 (71.4%) an overt disseminated intravascular coagulation was detected (≥5 points according to the International Society on Thrombosis and Haemostasis criteria) (Taylor et al., 2001; Toh and Hoots, 2007; Li et al., 2020).

Furthermore, high rate of deep venous thrombosis, complicated by lung embolism, has been reported in COVID-19 patients as well as myocardial infarction, stroke and critical limb ischemia (Bikdeli et al., 2020).

Moreover, in COVID-19 patients the cytokine storm, as an expression of exaggerated host immune system response, was characterized by very high level of erythrocyte sedimentation rate (ESR), C Reactive Protein (CRP), Tumor necrosis factor (TNF α), interleukins: IL-1β, IL-1RA, IL-2, (Li et al., 2020), IL-6, IL-7, IL-8, IL-9, IL-10, granulocyte-colony stimulating factor (GCSF), interferon gamma-induced protein-10 (CXCL10), monocyte chemoattractant protein-1 (MCP-1), macrophage inflammatory proteins 1 (MIP1) (Zhang J. et al., 2020), fibroblast growth factor (FGF), platelet derived growth factor (PDGF), vascular endothelial growth factor (VEGF) (Huang et al., 2020).

These data together with overt shock symptoms, such as feeble peripheral pulses and extremities pallor, with no overt hypotension, accompanied by metabolic acidosis in COVID-19 critical patients suggested a microcirculation dysfunction.

Therefore, investigations on microvascular and endothelial injury may have a fundamental role for explanation of pathophysiological mechanisms of COVID-19 clinical course and for development of new treatments for COVID-19 patients, to reduce the number of those who will need intensive care units. The endothelial injury, indeed, appears to be the key pathophysiologic factor leading patients to a multi organ failure (MOF) and even to death (Li et al., 2020).

The aim of the Italian Society of Clinical Hemorheology and Microcirculation is to stimulate the researchers to highlight the role of the microcirculatory dysfunction as the leading pathophysiologic factor of this new and unknown disease.

## Ace 2 Receptor Role

In the most severe forms, COVID-19 infection begins with lung damage. The reported data indicate alveolar damage, such as oedema or exudates, diffuse thickening of the alveolar wall and formation of hyaline membranes, pneumocyte hyperplasia, inflammation with multinucleated giant cell formation and macrophages infiltrating the air spaces (Barton et al., 2020; Gritti et al., 2020; Hanley et al., 2020; Tian et al., 2020; Xu et al., 2020; Zhou P. et al., 2020). Moreover, virus particles have been isolated in respiratory as well as fecal and urine specimen (Wang W. et al., 2020; Zhu et al., 2020). We do not know whether COVID-19 virus can directly target organs other than the lung; however, several reports describe that COVID-19 patients presented “spleen atrophy, hilar lymph nodes necrosis, focal hemorrhage in the kidney, enlarged liver with inflammatory cell infiltration, oedema, and degeneration of the neurons in the brain” (Chen G. et al., 2020; China National Health Commission, 2020; Li et al., 2020). It is interesting that a role could be played by the endothelial angiotensin-converting enzyme 2 receptor (ACE 2) localized in the lungs and other organs (Saxena, 2020). Indeed, the COVID-19 infects cells linking to the ACE 2 receptor which is expressed in lung and bowel epithelial cells, as well as in endothelial cells, cardiac cells, kidney tubular cells, neurons, alveolar monocytes and macrophages.

Patients affected by structural heart disease show increased ACE 2 expression, thus putatively representing a class of subjects exposed to a higher risk of developing critically ill condition (Chen L. et al., 2020; Sun et al., 2020; Zhou F. et al., 2020). Interestingly, hypertension and cardiovascular diseases were among the most prevalent comorbidities both in China and Italy (Magrone et al., 2020; Yanga et al., 2020).

In its turn, binding of SARS-CoV-2 to ACE 2 leads to its down-regulation as the virus uses the ACE 2 receptor for internalization and consequent cell damage/death. Because ACE 2 participates to the feed-back regulation of the renin-angiotensin system by counteracting ACE 1 (Chamsi-Pasha et al., 2014; Geng et al., 2020), excess ACE 1-dependent angiotensin II production leads to noxious vasoconstrictive, proinflammatory and pro-oxidative effects on the patient's vascular system through angiotensin-receptor 1 (ATR1) stimulation not counterbalanced by the ACE 2-dependent favorable effects triggered by MasR/ATR2 activation (Moccia et al., 2020). Covid-19-induced ACE 1/ACE 2 imbalance also leads to the so-called ACE 1 “shedding” phenomenon at the pulmonary vascular level, eventually enhancing local inflammation, pro-coagulant state and capillary leakage, likely increasing susceptibility to SARS-CoV-2 in remote tissues (Leisman et al., 2020). These mechanisms have been reported in detail and discussed in an accurate paper, suggesting further studies (Moccia et al., 2020).

However, we can assume that COVID-19 as well as or differently from other viral diseases causes direct damage on endothelial cells (Jeffers et al., 2004; Wang and Cheng, 2020). Preliminary data, indeed, from the post-mortem analysis of a COVID-19 patient indicate the presence of viral elements within the endothelial cells in intestinal microcirculation (Varga et al., 2020).

## Cytokine Storm

As above reported, in COVID-19 patients several cytokines and inflammation parameters are significantly increased. In particular, there are increments in the values of IL-6 and vascular endothelial growth factor (VEGF) (Huang et al., 2020). This “cytokine storm” indicates very high production of cytokines facilitating the inflammation mechanisms. This condition has been detected in patients affected by ARDS, characterized by pro-coagulant state and disseminated intravascular coagulation as well as by deep venous thrombosis and consequent embolism, thrombocytopenia and critical limb ischemia (Zhang W. et al., 2020).

The increase of cytokines has been described in several pathophysiological conditions, including infectious diseases, rheumatic diseases, vascular disease and tumor immunotherapy (Irace et al., 2004; Andreozzi et al., 2007; Zhang W. et al., 2020). Moreover, the CS has been described in previous coronavirus pneumonia, such as severe acute respiratory syndrome (SARS) and Middle East respiratory syndrome (MERS), leading to acute lung injury, acute respiratory distress syndrome (ARDS) (National Heart Lung Blood Institute Working Group Report, 2010; Dashti-Khavidaki and Khalili, 2020; Zhang W. et al., 2020) and death (Channappanavar and Perlman, 2017; Chousterman et al., 2017). However, in severe COVID-19 patients, Huang et al. reported that the cytokines were higher than in SARS and MERS patients (Conti et al., 2020; Huang et al., 2020); in particular, IL-6 levels were the highest in severely ill patients affected by COVID-19 (Chen G. et al., 2020; Ruan et al., 2020; Wu et al., 2020; Zhang W. et al., 2020; Zhou F. et al., 2020).

To date we do not know how the CS is produced at least in some patients. However, IL-1β, IL-6, TNF-α, and VEGF are released under different conditions, inducing an increase in microvascular permeability, especially VEGF. This molecule has been related also to hypoxia-inducible factor 1, produced under hypoxic conditions. Therefore, we could suggest that tissue hypoxia could promote release of some molecules. In an *in vivo* model of brain hypoxia (Lapi et al., 2020) it has been reported a more than 10 fold increase of thromboxane (TxB2) from endothelial cells, compared to control rats, by mass spectrometry analysis: TxB2 is known to increase intravascular coagulation and induce vessel constriction. This mechanism, operative in experimental conditions, could be investigated in CONAD 19 patients.

The CS could increase microvascular permeability and induce intravascular coagulation with embolization of different organs, such as lung or brain or heart and produce MOF (Xiao et al., 2011; Takao and Miyakawa, 2015).

A classification model of the disease into three progressive steps was suggested by Siddiqi and Mehra (Siddiqi and Mehra, 2020), who differentiated three grades of severity according to the clinical symptoms, response to therapy and outcome. A few of COVID-19 patients would experience the last stage of the disease, characterized by a systemic hyperinflammation syndrome. In the third stage, the levels of systemic inflammation markers were the highest. Therefore, the critical point was the timing of anti-inflammation therapy to counteract the CS and to decrease the death rate of the disease (Siddiqi and Mehra, 2020; Zhang W. et al., 2020).

## Endothelial Dysfunction

In sepsis red blood cells become less deformable and more easily aggregate each other compromising microvascular blood flow perfusion (Dellinger et al., 2008).

The hyperproduction of cytokines and chemokines, moreover, may induce increased activity of neutrophils, monocytes, and macrophages mobilization. Activated neutrophils and monocytes adhering to endothelial cells release reactive oxygen-derived free radicals that increase the damage to endothelium with impairment of endothelial barrier (Turer et al., 2008; Goldenberg et al., 2011; Hotchkiss et al., 2013; Carow and Rottenberg, 2014; Letsiou et al., 2015; Shalova et al., 2015; Weber et al., 2015).

Moreover, inflammatory cytokines, such as TNF-α, IL-1β, and IL-6 induce the synthesis of acute phase proteins by the liver, including fibrinogen (Mackiewicz et al., 1991), thereby producing a pro-coagulant state, at least in the venous circulation, and may also contribute to the increased arrhythmic risk observed in COVID-19 patients (Lazzerini et al., 2020).

The resulting pro-adhesive and prothrombotic effects stimulate a further adhesion of leukocytes and platelets to the vascular endothelium, causing vascular micro-thrombosis, capillary plugging and greater impairment of capillary flow (Levi et al., 1994; Vincent et al., 2009; Di Giandomenico et al., 2014). Activation of inducible Nitric Oxide Synthase in macrophages and other cells during viral sepsis could cause higher release of Nitric Oxide (NO), with consequent vasodilation and reduced systemic arterial blood pressure, with decreased response of vascular smooth muscle cells to nor-adrenergic stimulation (Fleming et al., 1990; Thiemermann and Vane, 1990). NO release by eNOS is known to contribute to arteriolar dilation and anti-platelet aggregation and adhesion to vascular wall cells, but in inflammation, hypoxia and endothelial dysfunction eNOS may be partially inhibited, with decrease of NO (Fleming, 2010). We detected significant decrease in eNOS expression in cerebral hypoperfusion-reperfusion injury at the end of the observations (Lapi et al., 2020). In lung hypoxia-reoxygenation injury, it has been suggested that eNOS-derived NO would induce an early protective effect against the organ damage, while iNOS-derived NO could have a late detrimental role, facilitating lipid peroxidation and apoptosis (Rus et al., 2010).

**Figure 1:**
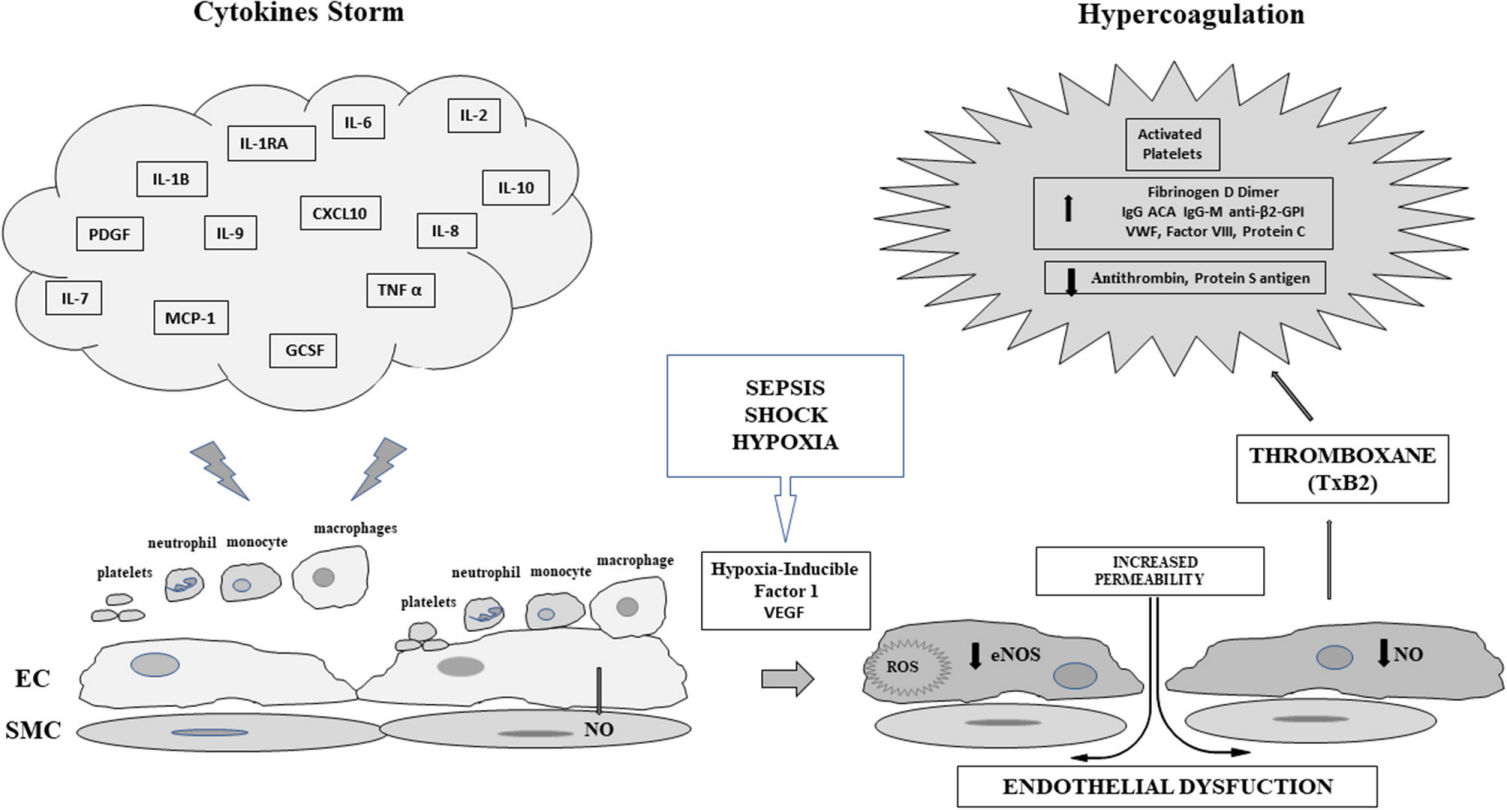
Scheme of the relationships between Cytokine Storm and Sepsis induced endothelial dysfunction in COVID-19: EC, Endothelial Cell; SMC, vascular smooth muscle cell; IL-1β, Interleukin 1-β; IL-1RA, Interleukin-1 receptor antagonist; IL-2, Interleukin-2; IL-6, Interleukin-6; IL-7, Interleukin-7; IL-8, Interleukin-8; IL-9, Interleukin-9; IL-10, Interleukin-10; PDGF, Platelet derived growth factor; CXCL10 C-X-C motif chemokine 10 or interferon gamma induced protein 10; MCP-1, Monocyte chemoattractant protein-1; TNF α, Tumor necrosis factor α; GCSF, Granulocyte colony stimulating factor; ROS, reactive oxygen species VEGF, Vascular Endothelial growth factor; NO, Nitric Oxide; eNOS, Endothelial Nitric Oxide Synthase; VWF, von Willebrand factor; IgG ACA, Immune globulin G Anticardiolipin Antibody; IgG-M anti-β2-GPI, Immune globulin G and M β2 glycoprotein Antibody.

Whether microvascular alterations are specifically due to COVID-19 or are an effect of the inflammatory pattern is not known (Bikdeli et al., 2020). A pro-coagulant state and systemic inflammatory response syndrome have been already observed in other viral infections (Borges et al., 2014; Ramacciotti et al., 2019; Smither et al., 2019; Mehta et al., 2020). Finally, elevated level of fibrinogen, D dimer, Protein C (Bikdeli et al., 2020) as well as IgG anti-cardiolipin antibodies (ACA) and anti-beta2-glycoprotein I (anti-β2-GPI) have been found in the most severe patients (Zhang Y. et al., 2020) with concurrent massive elevation of VWF antigen and activity, accompanied by an increase of Factor VIII (Escher et al., 2020). Moreover, a reduction of antithrombin III and protein S antigen has also been observed (Panigada et al., 2020). Therefore, the concrete mechanisms for coagulopathy are not known; in Figure 1 we present a scheme of the relationships among the CS, endothelial dysfunction and pro-coagulant state due to sepsis.

The data above reported indicate that there are no direct studies on microcirculation in COVID-19 patients. The reason is easily understood: the COVID-19 pandemic has hit the Health Systems of many Countries. The urgent need to find therapeutic solutions to contain the pandemic spread absorbed all the physical and mental resources of those who were on the front lines fighting against a disease totally new and unknown. It is evident from the data reported that COVID-19 targets the microcirculation. The microvascular failure is the fundamental pathophysiological mechanism leading the most severe patients to death.

It is reasonable to suggest that the virus directly attacks microvascular endothelial cells, but it is not known whether endothelial dysfunction can be considered as an event connected to the viral invasion or secondary to the cytokine storm. Probably both hypotheses come true simultaneously.

The virus infects endothelial cells by binding the ACE-2 receptor and using it for internalization, thus producing direct cell damage. In addition, an endothelial cell damage takes place also indirectly as the down-regulated ACE-2 receptor is no longer able to physiologically act in an inhibitory way on the ACE-induced production of angiotensin II.

Moreover, the inflammatory burden associated with the “cytokine storm,” which is the expression of an exaggerated response of innate immunity in its turn likely due to a late or ineffective response of adaptive immunity, is the final contributor to the damage: the consequent dysfunction of the microcirculation becomes the pathogenetic protagonist of SARS-CoV-2 infection.

## Conclusions

We are interested to implement the microvascular studies and the efforts to improve our knowledge regarding the microcirculation role in this disease. However, it is not so easy to evaluate microcirculation in critically ill patients. The bed side assessment in COVID-19 patients appears complex due to the prevention of the contagion for the health care personnel. Therefore, there is an urgent need for a reliable diagnostic equipment to assess the function of microcirculation in septic patients.

In conclusion, the COVID-19 represents one of the biggest challenges to the World Health, because disease is running fast across Countries. We hope that the efforts of all scientists and researchers will be effective in the contrast to COVID-19.

## Author Contributions

AC, RM, and PC planned and wrote and discussed the paper. PLC, AG, and GC wrote and discussed the paper. MB, RL, AM, and MR discussed and revised the paper. All authors contributed to the article and approved the submitted version.

## Conflict of Interest

The authors declare that the research was conducted in the absence of any commercial or financial relationships that could be construed as a potential conflict of interest.
